# Which symptoms pose the highest risk in patients calling for an ambulance? A population-based cohort study from Denmark

**DOI:** 10.1186/s13049-021-00874-6

**Published:** 2021-04-20

**Authors:** Stine Ibsen, Tim Alex Lindskou, Christian H. Nickel, Torben Kløjgård, Erika Frischknecht Christensen, Morten Breinholt Søvsø

**Affiliations:** 1 Centre for Prehospital and Emergency Research, Aalborg University and Aalborg University Hospital, Aalborg, Denmark; 2 Emergency Department, University Hospital Basel, University of Basel, Basel, Switzerland; 3 Prehospital Emergency Medical Services, North Denmark Region, Aalborg, Denmark

**Keywords:** Emergency medical services, Emergency medicine, Diagnosis, Mortality, Risk

## Abstract

**Background:**

Emergency medical service patients are a vulnerable population and the risk of mortality is considerable. In Denmark, healthcare professionals receive 112-emergency calls and assess the main reason for calling. The main aim was to investigate which of these reasons, i.e. which symptoms or mechanism of injury, contributed to short-term risk of death. Secondary aim was to study 1–30 day-mortality for each symptom/ injury.

**Methods:**

Historic population-based cohort study of emergency medical service patients calling 112 in the North Denmark Region between 01.01.2016–31.12.2018. We defined 1-day mortality as death on the same or the following day. The frequency of each symptom and cumulative number of deaths on day 1 and 30 together with 1- and 30-day mortality for each symptom/mechanism of injury is presented in proportions. Poisson regression with robust variance estimation was used to estimate incident rates (IR) of mortality with 95% confidence intervals (CI), crude and age and sex adjusted, mortality rates on day 1 per 100,000 person-year in the population.

**Results:**

The five most frequent reasons for calling 112 were “chest pain” (15.9%), “unclear problem” (11.9%), “accidents” (11.2%), “possible stroke” (10.9%), and “breathing difficulties” (8.3%). Four of these contributed to the highest numbers of deaths: “breathing difficulties” (17.2%), “unclear problem” (13.2%), “possible stroke” (8.7%), and “chest pain” (4.7%), all exceeded by “unconscious adult – possible cardiac arrest” (25.3%). Age and sex adjusted IR of mortality per 100,000 person-year was 3.65 (CI 3.01–4.44) for “unconscious adult – possible cardiac arrest” followed by “breathing difficulties” (0.45, CI 0.37–0.54), “unclear problem”(0.30, CI 0.11–0.17), “possible stroke”(0.13, CI 0.11–0.17) and “chest pain”(0.07, CI 0.05–0.09).

**Conclusion:**

In terms of risk of death on the same day and the day after the 112-call, “unconscious adult/possible cardiac arrest” was the most deadly symptom, about eight times more deadly than “breathing difficulties”, 12 times more deadly than “unclear problem”, 28 times more deadly than “possible stroke”, and 52 times more deadly than “chest pain”. “Breathing difficulties” and “unclear problem” as presented when calling 112 are among the top three contributing to short term deaths when calling 112, exceeding both stroke symptoms and chest pain.

## Background

The utilization of emergency medical services (EMS) has increased during the last decades and continues to do so in several high income countries [1–6]. EMS patients are a vulnerable population and the risk of mortality is considerable [7]. EMS focus on potential life-threatening and time-critical conditions such as cardiac arrest, respiratory failure, trauma, acute coronary syndrome, and stroke, named the First Hour Quintet [8].

EMS care must be initiated with little information about the patient’s medical history. In addition, EMS patients present with a variety of symptoms or clinical signs, not always clearly indicating the severity of the condition.

Emergency calls in Denmark are answered by the police and since 2011, calls of medical nature are forwarded to healthcare professionals, who assess the severity according to the patients’ symptoms or mechanism of injury, and determine the appropriate response with the help of the criteria-based dispatch, the Danish Index for Emergency Care (Danish Index). The system was original developed in King County Washington in 1990 [9] and further modified into Scandinavian context [10–12]. The Danish Index is not a fixed protocol, but a decision-support tool to categorise each emergency call into a main criterion e.g. chest pain and an urgency level for the ambulance response.

These initial symptoms or mechanism of injury at the first patient contact seem to carry important prognostic information which might inform the care of the patient. However, outcomes have been investigated in isolated symptoms only, such as chest pain [13] and breathing difficulty [14].

The main aim of this present study was to investigate the symptoms or mechanism of injury contributing most to short-term mortality among patients calling the emergency number (112). Furthermore, we investigated 1- and 30-day mortality for individual Danish Index criteria.

## Methods

### Study design

Historic population-based cohort study based on routinely collected healthcare data from the years 2016–2018. We followed The Strengthening the Reporting of Observational Studies in Epidemiology (STROBE) Statement [15].

### Setting

Emergency calls (112-calls) that relates to a medical emergency, is forwarded to an Emergency Medical Coordination Centre (EMCC) where healthcare professionals answer the call. The healthcare professionals are registered nurses, peer-trained to handle emergency calls and to register relevant data (including Danish Index). The healthcare professionals have the option to advise or refer the patient, as well as to dispatch an ambulance [16]. The healthcare professionals use the Danish Index to assess the main reason for calling among a total of 37 criteria, such as for example unconscious adult and accidents. Each criterion is subdivided into five urgency levels (A-E). Level A describes life threatening conditions or potential life threatening conditions, level B is urgent, but not life threatening conditions, level C is non-urgent conditions that require an ambulance, level D is non urgent conditions requiring supine patient transport, and level E is conditions requiring medical advice only [17]. Each of the five Danish healthcare regions have an EMCC. This study included data from the North Denmark Region, with both urban and rural areas and about 590,000 inhabitants, corresponding to 10% of the Danish population [18]. The Danish healthcare system including EMS is free to all citizens [16].

### Participants

We defined emergency call patients as patients who had been in contact with the EMCC between 01.01.2016–31.12.2018, including patients with more than one contact during the study period and patients advised or referred over the phone, without a dispatched ambulance. Patients brought to hospital by planned transportation were not included in this study. Patients without registered Danish Index and/or civil registration number were excluded from the study. Patients from other regions or patients without residence in Denmark were excluded from the mortality analysis as date of death was only available for patients with fixed abode in the North Denmark Region.

### Variables and outcome measurements

The main variables included number of emergency calls from 01.01.2016–31.12.2018 and the distribution of Danish Index criteria assessed by the healthcare professionals. We reported the distribution of Danish Index criteria. Logistic data on the ambulance run and Danish Index criteria were retrieved from the Logis CAD system *(Logis Solutions A/S, Nærum, Denmark).* Date of death, age, and sex were obtained from the Danish Civil Registration System [19].

Primary outcome was mortality in terms of numbers and proportions of deaths at day 1 and day 30 after the emergency call among all deaths in the 112-patient population, together with incidence rates of mortality in the population for the top five criteria with the highest number of cumulative deaths. The choice of focusing on the top five was arbitrary. Secondary outcomes were 1–30 day-mortality for each Danish Index criteria. Time of death is registered by date without time of day in the Danish Civil Registration System. We therefore defined 1-day mortality as death within the same day as the emergency call or the following day. Patients registered with time of death before the emergency call were excluded for the mortality analysis. A 30-day exclusion period, for the mortality analysis, for patients with more than one emergency call were implemented. As such, if a patient had more than one call within 30 days, only the last call was included.

### Statistics

Descriptive analyses were performed by use of numbers and percentages for the distribution of Danish Index criteria, 1- and 30-day mortality. Poisson regression with robust variance estimation were used to estimate incidence rates (IR) of mortality with 95% confidence interval (CI), and IR adjusted for age and sex. 1–30-day mortality for the five criteria contributing most to all deaths were visualised using Kaplan-Meyer curves. Data was anonymized prior to analysis.

Stata/MP 15.1 *(StataCorp LLC, Texas, United States of America)* was used for all statistical analysis.

### Ethics

The study was registered at the North Denmark Region (2019–112) and the Danish Patient Safety Authority approved the study (3–3013-1675/3).

## Results

A total of 98,244 emergency calls was registered from 01.01.2016–31.12.2018 in the North Denmark Region. The number of emergency calls increased with 10% from 31,967 in 2016 to 35,209 in 2018. No identifiable civil registration number was available for the patient in 8659 cases (8.8%). Time of death was noted before the emergency call in 17 cases, and 18 emergency calls were excluded due to duplicate registrations. In 4652 (4.7%) of the emergency calls, the Danish Index criterion was missing or incomplete. The number of missing Danish Index criteria decreased in the study period from 1955 cases (6.7%) in 2016 to 966 cases (3.0%) in 2018.

**Fig. 1:**
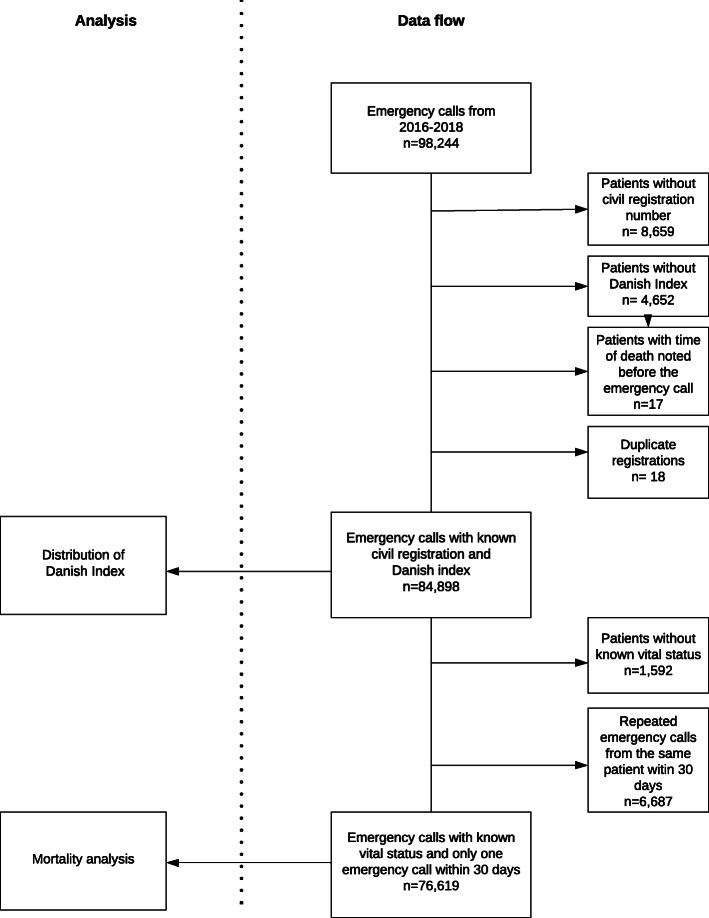
Data flowchart illustrates the exclusion and inclusion of emergency call patients

As such, we included 84,898 emergency call patients. For the mortality analysis we excluded 8316 (9.7%) emergency calls, due to unknown vital status (1592 cases) and repeated emergency calls within 30 days (6687 cases). Figure 1 illustrates data flow.

**Fig. 2:**
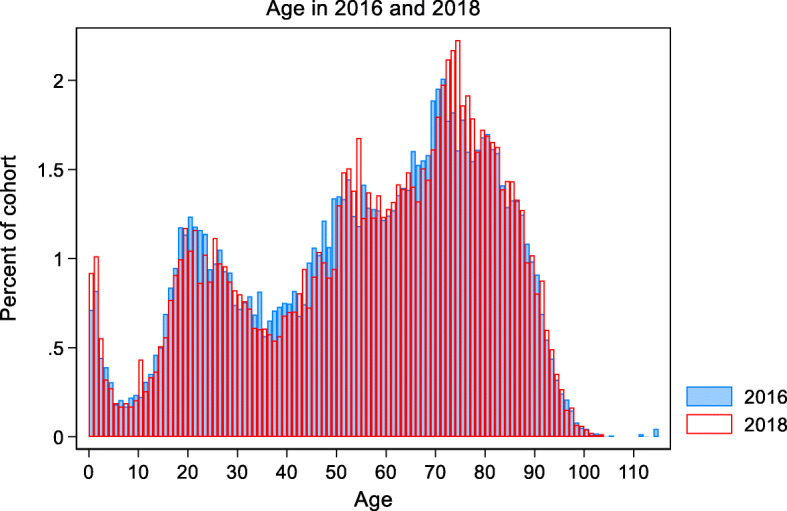
Age (in years) distribution among emergency callers in 2016 and 2018 (*n* = 85,615)

The proportion of women was 46.3% and the mean age was 55.3 years. Patients covered all age groups with four distinct peaks among the youngest children as well as patients in their 20s, 50s and 70s (see Fig. 2).

### Distribution of Danish index criteria

The most frequently recorded Danish Index criteria corresponding to nearly every seventh call, was “chest pain – possible heart disease” (from here on referred to as “chest pain”), followed by “unclear problem”, “accidents”, “possible stroke”, and “breathing difficulties”. The five most frequent Danish Index criteria constituted 58% of all emergency calls. Table 1 shows the distribution of Danish Index criteria, and sex and age.

**Table 1:** Distribution of Danish Index criteria in numbers (n) and percentages (%), proportion of women in n and % and mean age with standard deviation (SD)

Danish index criteria	Number ***n***(%)	Women%	Age mean (SD)
Chest pain	13,586(15.9)	43.4	62.0(18.2)
Unclear problem	10,171(11.9)	46.3	60.8(22.2)
Accidents	9549(11.2)	45.3	52.4(26.6)
Possible stroke	9345(10.9)	47.8	66.4(18.5)
Breathing difficulties	7111(8.3)	50.1	64.8(21.7)
Pain in the extremities – wounds – fractures – small injuries	5716(6.7)	51.9	57.7(25.5)
Abdominal pain - back pain	4930(5.8)	49.5	50.5(21.3)
Traffic accident	4181(4.9)	42.6	39.5(20.8)
Seizures	3677(4.3)	44.2	31.7(25.5)
Alcohol - poisoning – overdose	3236(3.8)	43.2	35.4(17.4)
Unconscious adult/ possible cardiac arrest	2813(3.3)	43.0	66.1(20.2)
Bleeding - not traumatic	1674(1.9)	40.9	63.8(20.9)
Diabetes	1506(1.8)	49.9	53.3(19.1)
Psychiatry – suicidal	1223(1.4)	50.5	39.5(17.1)
Allergic reaction	853(1.0)	54.9	45.6(22.4)
Sick child	820(1.0)	44.9	3.1(9.1)
Headache	787(0.9)	54.0	48.1(21.7)
Violence – abuse	544(0.6)	23.4	34.4(13.5)
Ear - nose – throat	496(0.6)	45.8	60.7(23.6)
Burns - electric injury	490(0.6)	38.6	37 (23)
Fever	485(0.6)	38.9	52.2(27.6)
Gynaecology – pregnancy	461(0.5)	99.8	30.2(9.4)
Urinary system	379(0.4)	16.6	65.8(21.5)
Foreign body in airways	229(0.3)	50.2	39.9(34.6)
Birth	126(0.2)	100.0	29.0(5.6)
Eye	112(0.1)	32.1	41.7(23.5)
Unconscious child (before puberty)	94(0.1)	52.1	6.6(15.3)
Animal bite - insect bite	86(0.1)	38.4	42.3(23.0)
Hypothermia – Hyperthermia	59(0.1)	42.4	61.8(26.7)
Drowning	51(0.1)	41.2	44.2(22.2)
Poisoning in children	37(0.0)	37.8	6.9(13.9)
Chemicals – gasses	36(0.0)	22.2	30.2(22.7)
Catastrophe - big accidents	16(0.0)	64.3	57.6(19.9)
Skin and rash	12(0.0)	41.7	58.8(17.3)
Diving accident	7(0.0)	28.6	38.3(23.1)
All	84,898(100.0)	46.3	55.3(24,5)

### Mortality

A total of 2997 patients (3.9%) died the same day as the emergency call or the following day and 4571 patients (6.8%) died within 30 days from the emergency call in the period from 2016 to 2018. The number and percentages among all deaths in 112-patients as well as the 1- and 30-day mortality for each criterion is shown in Table 2.

**Table 2:** Number of deaths on day 1 and day 1–30 for Danish Index criteria (n) and percentages (%) (row percentages) and for all deaths (column percentages)

Danish Index criteria	***N***	Day 1 = same or following day after 112 call		Day 30 after 112 call	
		Deaths day 1 Numbers (%)	Percentage of all deaths	Deaths day 1–30 cumulative Numbers (%)	Percentage of all deaths
Unconscious adult/ possible cardiac arrest	2657	1146(43)	38.1	1335(50.3)	25.3
Breathing difficulties	6158	317(5.1)	10.5	907(14.8)	17.2
Unclear problem	9212	292(3.2)	9.7	697(7.6)	13.2
Possible stroke	8632	133(1.5)	4.4	460(5.6)	8.7
Chest pain	12,071	83(0.7)	2.8	246(2.1)	4.7
Accidents	8879	54(0.6)	1.8	216(2.4)	4.1
Pain in the extremities - wounds - fractures - small injuries	5267	16(0.3)	0.5	140(2.6)	2.7
Abdominal pain - back pain	4431	44(0.9)	1.5	123(2.8)	2.3
Bleeding - not traumatic	1472	30(2.1)	0.9	103(6.9)	1.9
Psychiatry – suicidal	1029	53(5.2)	1.8	62(5.9)	1.2
Traffic accident	4019	41(1.0)	1.4	55(1.4)	1.0
Diabetes	1174	18(1.5)	0.6	49(4.2)	0.9
Seizures	3133	12(0.4)	0.4	48(1.6)	0.9
Fever	456	6(1.3)	0.2	27(5.9)	0.5
Alcohol **-** poisoning – overdose	2781	12(0.4)	0.4	19(0.7)	0.4
Headache	736	4(0.5)	0.1	16(2.2)	0.3
Foreign body in airways	219	8(3.6)	0.3	11(0.5)	0.2
Burns - electric injury	470	6(1.3)	0.2	10(2.1)	0.2
Urinary system	325	4(1.2)	0.1	10(3.1)	0.2
Unconscious child/ possible cardiac arrest	84	7(8.2)	0.2	7(8.2)	0.1
Drowning	43	7(16.3)	0.2	7(16.3)	0.1
Ear - nose – throat	410	1(0.2)	0.0	6(1.5)	0.1
Hypothermia - Hyperthermia	47	1(2.1)	0.0	4(8.5)	0.1
Violence – abuse	493	2(0.4)	0.1	4(0.8)	0.1
Allergic reaction	790	1(0.1)	0.0	2(0.3)	0.0
Gynaecology - pregnancy	426	0(0)	0.0	2(0.5)	0.0
Diving accident	7	1(14.3)	0.0	1(14.3)	0.0
Skin and rash	11	0(0)	0.0	1(9.1)	0.0
Sick child	773	1(0.1)	0.0	1(0.1)	0.0
Catastrophe - big accidents	12	0(0)	0.0	0(0)	0.0
Animal bite - insect bite	75	0(0)	0.0	0(0)	0.0
Poisoning in children	35	0(0)	0.0	0(0)	0.0
Birth	121	0(0)	0.0	0(0)	0.0
Chemicals – gasses	32	0(0)	0.0	0(0)	0.0
Eye	109	0(0)	0.0	0(0)	0.0
Total	**77,313**	**2997(3.9)**	**100**	**4571(6.8)**	**100**

The top five of criteria with the highest number and proportions of all deaths on day 1 and cumulative on day 1–30 was “unconscious adult/possible cardiac arrest”, “breathing difficulties”, “unclear problem”, “possible stroke”, and “chest pain”, respectively. These five symptoms with the highest number of deaths contributed to 69% of all deaths within 30 days from the emergency call.

### Mortality Danish Index criteria

Patients with Danish Index criteria “unconscious adult/ possible cardiac arrest” exhibited the highest IR mortality in the population among the top five of Danish Index criteria with the highest numbers of cumulative deaths, adjusted and unadjusted, followed by “breathing difficulties”, “unclear problem”, “possible stroke”, and “chest pain”. IR for mortality decreased with adjustments for age and sex but showed same pattern. IR for short-term mortality within the same or following day (=day 1) per person and per 100,000 person years were all significantly different, as there was no overlap of CI between the Danish Index criteria, demonstrating significant differences in IR of mortality for the five criteria, crude as well as age - and sex – adjusted, as shown in Table 3. In a population of 100,000 per year, 26.75 deaths can be expected on the same day or the day after the emergency call concerning “unconscious adult/ possible cardiac arrest”, and 3.65 when adjusted for age and sex. This is about eight times higher adjusted IR than “breathing difficulties”’ (0.45), 12 times higher than “unclear problem” (0.30), 28 times higher than “possible stroke” (0–13), and 52 times higher than “chest pain” (0.07). The adjusted rates showed an overall decline in mortality for all five symptoms; thus, the risk of death increases with higher age and was higher for men than women.

**Table 3:** Estimated crude and adjusted (by sex and age) 1-day mortality rates per 1 and per 100,000 person years with 95% CI for the top five Danish Index criteria

Danish Index Criteria	1-day mortality Per person year		1-day mortality Per 100,000-person year	
	Crude	Adjusted	Crude	Adjusted
	IR(95%CI)	IR(95%CI)	IR(95%CI)	IR(95%CI)
Unconscious adult/ possible cardiac arrest	157.82(151.07–164.86)	21.55(17.73–26.21)	26.75(25.6–27,9)	3.65(3.01–4,44)
Breathing difficulties	18.81(16.89–20.94)	2.69(2.20–3.30)	3,18(2.86–3.55)	0.45(0.37–0.56)
Unclear problem	11.53(10.29–12.91)	1.78(1.47–2.17)	1.95(1.74–2.19)	0.30(0.25–0.36)
Possible stroke	5.65(4.77–6.68)	0.79(0.62–1.00)	0.96(0.81–1.13)	0.13(0.11–0.17)
Chest pain	2.52(2.03–3.12)	0.39(0.30–0.51)	0.43(0.34–0.53)	0.07(0.05–0.09)

**Fig. 3:**
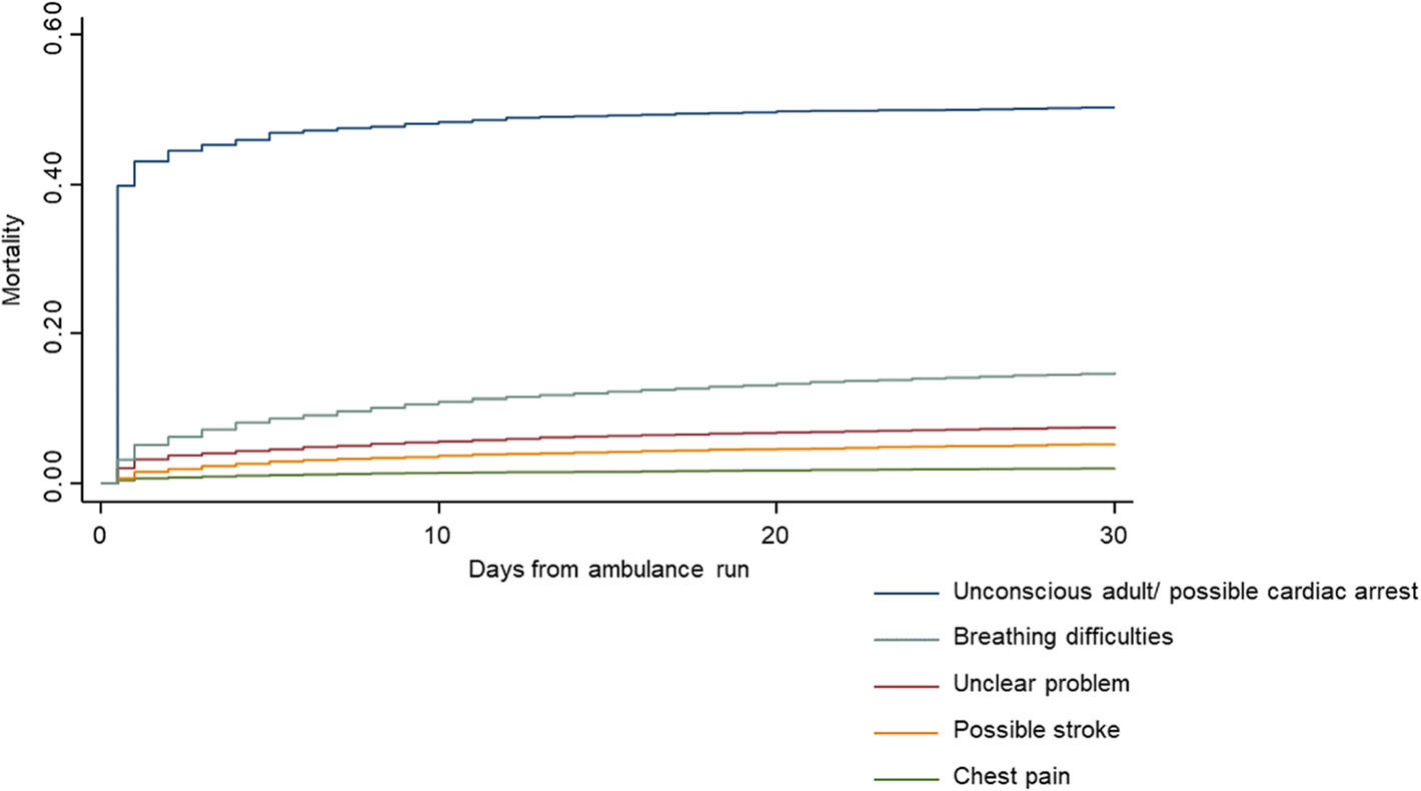
Mortality up to 30 days after an emergency call, among emergency ambulance service patients with the top five Danish Index criteria with the highest cumulative number of deaths

Kaplan-Meier estimate of mortality for the top five Danish Index with the highest number of deaths within 30 days from the emergency call is shown in Fig. 3. Kaplan-Meier estimates of mortality.

## Discussion

This historic population-based cohort study of patients calling for an emergency ambulance in the North Denmark Region showed that the top five of Danish Index criteria contributing most to the total number of deaths among 112-patients was “unconscious adult/possible cardiac arrest”, “breathing difficulties”, “unclear problem”, “possible stroke”, and “chest pain”. Equivalent results were demonstrated for age and sex adjusted IR estimates of short-term mortality, i.e. death on the same or the following day after the 112-call, on annual population level per 100,000 people. There were large differences between each of the five criteria, with IR mortality for “unconscious adult/possible cardiac arrest” more than 50-fold as high as for chest pain. These five conditions corresponded almost to the five most frequent criteria for calling 112. The only exception was “unconscious adult/possible cardiac arrest” which was much more seldom, whereas “accidents” was the third most frequent among the top five most frequent criteria.

A strength of this study was the availability of data and the opportunity to link registers, which enabled the follow-up and minimised the risk of information bias. Furthermore, the North Denmark Region covers mixed rural and urban areas and the free access to healthcare in Denmark minimises the risk of selection bias. A weakness of the study was the missing civil registration number for 8.7% of the emergency calls and the missing Danish Index in 4.9% of the calls. The number of missing Danish Index criteria decreased in the study period from 6.7% in 2016 to 3.0% in 2018. The Danish Index was implemented in 2011 and staff training might explain the increase in registrations. However, it cannot be ruled out that these excluded calls may represent a certain group of patients and thereby bias the results. They could represent patients with similar symptoms or potentially patients who are more, or less severely ill or injured, and thereby affect the results in either direction. However, it is a low percentage of the overall large number of included patients and is likely to have a minor impact on the results. Furthermore, the proportion of missing data was similar or even lower than in previous studies, where missing data due to unknow civil registration number reached 17.8% [1] and missing data due to unknow civil registration and missing Danish Index reached 45% [20].

Patients with repeated emergency calls within 30 days only had the last contact included for the mortality analysis, which may have resulted in underestimation as well as overestimation of mortality for some of the Danish Index criteria, as the initial call might be due to another symptom or mechanism of injury than the last call during the 30-day period. However, as only 8% of the patients had repeated runs, we assess this to have little influence. The repeated users are however a special group, that would benefit from further investigation.

This study included patient symptoms or mechanism of injury in relation to the Danish Index and thereby each patient is only registered with one criteria as the main reason for calling, although patients may present with more than one symptom [21]. This problem is well known, and similar to other studies on presenting main symptom [22, 23].

Our study confirms that the First Hour Quintet, i.e. cardiac arrest, respiratory failure, trauma, acute coronary syndrome, and stroke are serious conditions associated with high mortality, and we found that four of these were among the top five contributing to total mortality among 112-patients. “Accidents” was the third most frequent reason for calling 112, and number six contributing to deaths among 112-callers, with 1- and 30-day mortality of 0.6 and 2.4%, very similar to number five on our list, “chest pain” with 0.7 and 2.1%. Our study revealed that “unclear problem” is a high-risk symptom, as it had the third highest cumulative number of deaths within 30 days and was the second most frequent Danish Index criterion, and altogether making this the third most deadly symptom in 112-calls as reflected in the mortality IR, both crude and adjusted. This study showed that the percentages of all death was 3.9% for 1-day mortality and 6.8% for 30-day mortality. This is similar to results from a previous cohort study from Denmark based on data from 2012 to 2014, that demonstrated an overall mortality on 5.3% [24]. The adjusted mortality rates showed that the risk of mortality after a 112-call increases with higher age and that men had a higher mortality than women. We adjusted for the main effect by age and gender since mortality is related to both. However, there might still be residual confounding present, which could have been handled with interactions terms. In the present study grouping age in, for instance, 3 groups stratified into 5 symptoms and 2 genders would have produced 30 groups. This would result in a small number for each group and thereby increase the risk of wrong estimates and confidence intervals. Thus, we decided not to perform stratification/interaction analyses in this study. It would be relevant for future studies to thoroughly investigate the age and gender effect on mortality in larger datasets and for individual symptoms.

“Unclear problem” is usually assigned when the healthcare professional does not know the exact medical cause, a phenomenon which is already well-described for emergency departments. Likewise, the high mortality for “unclear symptom” corresponds to what have been previously described for non-specific symptoms presented in emergency departments [22, 25]. A Swedish study of EMS patients arriving at an emergency department, found that the risk of having an ambulance dispatched with low priority by the EMCC was almost doubled among patients with non-specific complaints compared to randomly selected patients matched for age and sex [26]. A Danish register-based study from the capital region in 2011–2013 found that 18% of emergency calls were categorized as unclear problem, and in calls assessed with emergency level B (without light and sirens), they found that unclear problem had a higher mortality than specific symptoms or problems [20]. Another study, covering 75% of the Danish population, elucidated potential preventable deaths due to the medical dispatch decisions by auditing medical records for the 152 EMS patients dying the same day as receiving an ambulance with urgency level B. They found only few preventable deaths, but among these, the most frequent criteria were “unclear problem” and “breathing difficulties” [7]. Thus, unclear problem in EMS seems to be a similar challenge as non-specific symptoms in the emergency departments.

Breathing difficulties has previously been demonstrated to be among the most common reasons for contacting EMS [4]. Likewise, the high mortality for breathing difficulties has previously been established, both in EMS and the emergency departments [21–23, 27]. A previous study assessed how well nurses, physicians, and patients agreed on an 11-point rating scales of breathlessness, and found an underestimation of breathlessness and respiratory function by nurses and physicians in the intensitive care units [28]. This may play a role in the challenging task for healthcare professionals at the EMCC to estimate the severity of this particular symptom. A recent Swedish study confirmed high mortality rates for patients with dyspnoea, and interestlingly found that 84% of the patients had previously suffered from dyspnoea and more than half showed more than two days delays from symptoms onset to EMS contact [29]. This shows that many patients with dyspnoa/breathing difficulties call for help as the condition has become unmanageable, which emphazises the vulnerability of these patients.

In 2012, the most frequently used Danish Index criteria were (1) *unclarified problem*, (2) *chest pain*, (3) *minor wounds and injuries* (4) *accidents* and (5*) breathing difficulties* [10]*,* similar to our study, except for “possible stroke” now on top five*.* Early recognition of stroke leads to faster response and improves time to hospital arrival [30, 31] which in turn improves the diagnosis and treatment. The increased awareness for symptoms of stroke, may explain the increase in healthcare professionals’ assessment of the symptom “possible stroke”. In Denmark there has been several campaigns to raise awareness of symptoms and risk factors of stroke.

Our study showed a four to six times lower risk of short-term mortality for the symptom “chest pain” when compared to the frequently recorded symptoms, i.e. “breathing difficulties” and “unclear problem”. Likewise, an Irish study found that patients having chest pain as presenting complaint when admitted to a hospital, was associated with a decreased risk of 30-day mortality with a odds ratio of 0.47. Whereas, the study demonstrated breathing difficulties to be associated with an increased risk of death, with an odds ratio of 1.8 [32].

Follow up on patients with Danish Index “unclear problem” and “breathing difficulties” in terms of conditions could provide valuable knowledge of which patients are most sensitive for possible adverse consequences or not being recognized early. We need to know more about the underlying conditions and identify the complaints that increases risk for adverse outcome. Investigation of vital signs and initial treatment in the ambulances could explain to what extent these patients were critically ill and in need of immediate treatment. Moreover, analysing communication, tone and anxiety in the speech during the calls by audio and/or video using communication analysis and artificial intelligence maybe useful. Recognition of time-critical conditions is import for patient outcome and the presenting symptoms carry valuable information of the risk of short-term mortality. The magnitude of “unclear problem” indicates the need for more research into this group of patients: on how they are handled on scene and in hospital; to which extent these patients are acutely ill requiring immediate help; whether patients with “unclear symptom” encompass terminal or old dying patients and/or socioeconomic vulnerable patients. This may be an overlooked and neglected patient group with potential for future improvements in the entire patient care pathway.

## Conclusion

This study showed that “breathing difficulties” and “unclear problem” as presented when calling 112-call are among the top three symptoms contributing to short term deaths when calling 112, only exceeded by possibly cardiac arrest. As number four and five we found “possible stroke” and “chest pain”. In terms of risk of death on the same day and the day after the 112-call, “unconscious adult/possible cardiac arrest” was by far the most deadly symptom, about eight times more deadly than “breathing difficulties”, 12 times more deadly than “unclear problem”, 28 times more deadly than “possible stroke” and 52 times more deadly than “chest pain”. When calling the emergency number for an ambulance, “unclear problem” bears risk of short-term death at similar level as other well-known severe organ-related complaints.

## Data Availability

As the study include sensitive patient information, restrictions apply to the availability of data that is not publicly available. However, researchers interested in the data can seek approval from the Danish Patient Safety Authority. Having obtained approval, researchers can request data from the Centre for Prehospital and Emergency Care, Aalborg Denmark.

## Abbreviations

EMS: Emergency medical services
Danish Index: Danish Index for Emergency Care
EMCC: Emergency Medical Coordination Centre
IR: Incidence rate
